# *Aspidosperma* (Apocynaceae) plant cytotoxicity and activity towards malaria parasites. Part II: experimental studies with *Aspidosperma ramiflorum* in vivo and in vitro

**DOI:** 10.1590/0074-02760150188

**Published:** 2015-11

**Authors:** Anna CC Aguiar, Ananda C Cunha, Isabela Penna Ceravolo, Regina A Correia Gonçalves, Arildo JB Oliveira, Antoniana Ursine Krettli

**Affiliations:** 1 Fundação Oswaldo Cruz, Centro de Pesquisas René Rachou, Belo Horizonte, MG, Brasil; 2 Universidade Federal de Minas Gerais, Faculdade de Medicina, Belo Horizonte, MG, Brasil; 3 Universidade Estadual de Maringá, Departamento de Farmácia, Programa de Pós-Graduação em Ciências Farmacêuticas, Maringá, PR, Brasil

**Keywords:** *Aspidosperma ramiflorum*, ethnopharmacology, antimalarials, *P. falciparum*, medicinal plants

## Abstract

Several species of *Aspidosperma* plants are used to treat diseases in the tropics, including *Aspidosperma ramiflorum*, which acts against leishmaniasis, an activity that is experimentally confirmed. The species, known as *guatambu*-yellow, yellow *peroba*, coffee-*peroba* and*matiambu*, grows in the Atlantic Forest of Brazil in the South to the Southeast regions. Through a guided biofractionation of* A. ramiflorum *extracts, the plant activity against* Plasmodium falciparum *was evaluated in vitro for toxicity towards human hepatoma G2 cells, normal monkey kidney cells and nonimmortalised human monocytes isolated from peripheral blood. Six of the seven extracts tested were active at low doses (half-maximal drug inhibitory concentration < 3.8 µg/mL); the aqueous extract was inactive. Overall, the plant extracts and the purified compounds displayed low toxicity in vitro. A nonsoluble extract fraction and one purified alkaloid isositsirikine (compound 5) displayed high selectivity indexes (SI) (= 56 and 113, respectively), whereas compounds 2 and 3 were toxic (SI < 10). The structure, activity and low toxicity of isositsirikine in vitro are described here for the first time in *A. ramiflorum*, but only the neutral and precipitate plant fractions were tested for activity, which caused up to 53% parasitaemia inhibition of *Plasmodium berghei* in mice with blood-induced malaria. This plant species is likely to be useful in the further development of an antimalarial drug, but its pharmacological evaluation is still required.

Malaria, one of the most prevalent parasitic diseases in the world, still causes a high morbidity and is responsible for approximately 600,000 deaths yearly worldwide mainly due to *Plasmodium falciparum *(WHO 2014). This increasing global importance is a result of the spread of drug-resistant parasites, the current limitations of vector control and the absence of an effective vaccine. Treatment remains the main strategy for malaria control and new drugs are required (Ridley 2002, de Ridder et al. 2008, WHO 2014).

Popular medicine remains an important source for malaria treatment using plant remedies in the endemic areas of African sub-Saharan countries (Willcox & Bodeker 2004, Bourdy et al. 2007, Adebayo & Krettli 2011) and in Latin America (Oliveira et al. 2009, 2015),^2015^). The use of plants in the search for new antimalarials is also influenced by the fact that two important antimalarials, quinine and artemisinin, originated from the *Cinchona* species native to the Peruvian Amazon (White 2008) and the*Artemisia annua *native to China (Cui & Su 2009), respectively.

The activities of several plant species used against fever and malaria in Brazil have been tested against malaria parasites after fractionation and chemical characterisation (Krettli et al. 2001, 2009, Frausin et al. 2015). Plants of the *Aspidosperma* genus tested in vitro and in vivo were active, like the compounds from the barks of *Artemisia vargasii*,*Artemisia ulei* and *Artemisia desmanthum* (de Andrade-Neto et al. 2007, Rocha e Silva et al. 2012, Torres et al. 2013), as well as *Artemisia nitidum*, which is used in the Amazon against fever and malaria (Coutinho et al. 2013), and *Artemisia olivaceum*, a plant from South Brazil (Aguiar et al. 2012).

The extracts and compounds purified from *Aspidosperma ramiflorum*(Apocynaceae), known as *gua- tambu*-yellow and yellow*peroba*, *matiambu*, *matambu*,*guatambu-grande*,* peroba-café* and*pequiá*, are now being studied. The species is native in the Atlantic river forests of Brazil from the South Region [states of Santa Catarina and Paraná (PR)] to the Southeast Region [states of Rio de Janeiro and Minas Gerais (MG)]. Several plant fractions are active against *Leishmania amazonensis*parasites. The compounds responsible for the activity are the monoterpenoid indole alkaloids (Cunha et al. 2012), which are present in large amounts in the plant and were again isolated (Fig. 1) and studied for activity against *P. falciparum* in vitro. Some extracts were tested against malaria in mice with*Plasmodium berghei*.

**Fig. 1:**
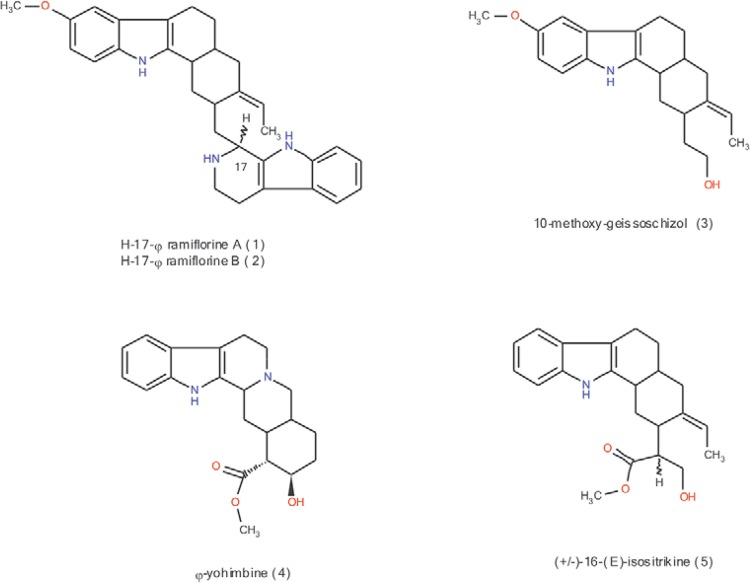
major monoterpenoid indole alkaloids from *Aspidosperma ramiflorum*. Drawing, displaying and characterising the chemical structures, substructures and reactions were performed using Marvin 5.4.1.1, 2011 (ChemAxon) (chemaxon.com).

## MATERIALS AND METHODS

*Ethics*- The use of laboratory animals was approved by the Oswaldo Cruz Foundation (Fiocruz) Ethical Committee for Animal Use (CEUA LW23/13).

*A. ramiflorum (Müll. Arg.) plant material* - The plant materials were collected in July 2010 in Maringá (PR) at the forest garden (51º30'54ºW 22º30'S) at an altitude of 556 m by Dr Luís Teixeira Mendes. Permission for the collection was provided by the System Authorisation and Information on Biodiversity, Brazilian Institute of Environment and Renewable Natural Resources (registration 5003641). The species was identified by Prof Washington Marcondes Ferreira, Department of Biology, University of Campinas (state of São Paulo, Brazil), and a voucher (HUEM 20501) was deposited at the State University Herbarium, Maringá. An adult specimen may reach 20-30 m in height (sementesdopantanal.dbi.ufms.br/entrada.php?inf= 1&opcao= 1&id= 1115).

*Extraction of plant materials* - The collected *A. ra- miflorum* materials (stem bark 2 kg and leaves 0.340 g) were dried in a circulating air oven, ground and subjected to extraction by maceration with methanol for seven days. The organic solvent was evaporated at 40°C under rotation and reduced pressure. The crude extract was lyophilised and subjected to acid base fractionation, as previously described (Marques et al. 1996, Cunha et al. 2012). After a complete partition of the plant stem bark methanolic crude extract (138 g), four alkaloid rich fractions were obtained: the acid, the neutral precipitate (NP), the neutral and the basic fractions, which were all concentrated under reduced pressure at 40ºC, lyophilised and provided for biological testing at the René Rachou Research Centre, Fiocruz (Belo Horizonte, MG).

**Fig. 2:**
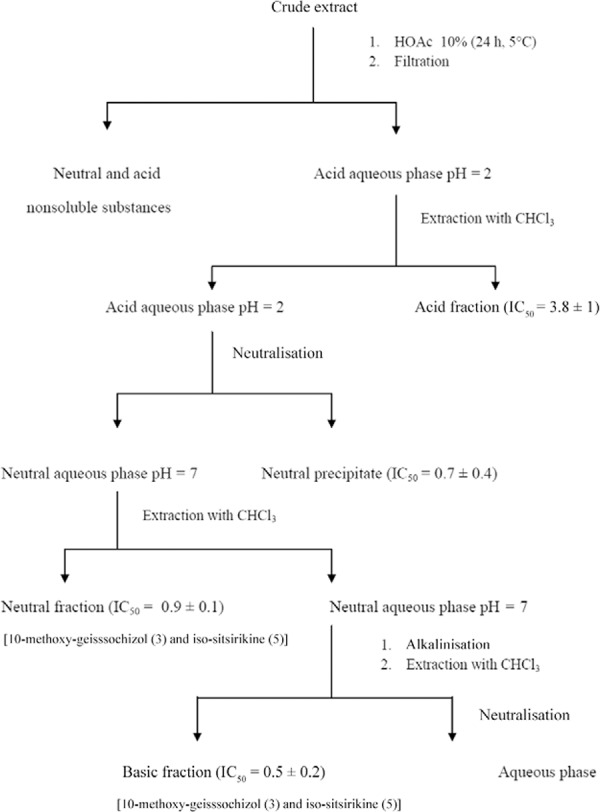
flowchart fractionation of *Aspidosperma ramiflorum*using acid base crude extract from stem bark of the plant. IC50: half-maximal drug inhibitory concentration.

A crude extract of the plant leaves (20.4 g) was subjected to a simplified acid-base partition employing the same solvents (Tanaka et al. 2007). Two alkaloid rich fractions were obtained (the acid fraction and the basic fraction) and were concentrated under reduced pressure at 40ºC for solvent evaporation and then lyophilised (Cunha et al. 2012) (Fig. 2).

The alkaloid extracts from the stem barks and the leaves were analysed by thin-layer chromatography (TLC) on a silica gel 60 GF_254_ and developed with CHCl_3_/MeOH 92:8 in an NH_3_ atmosphere with the*p*-anisaldehyde reagent followed by heating to 105ºC for 2-4 min.

*Electrospray ionisation mass spectrometric analyses (ESI-MS) -*Samples from the extracts of the *A. ramiflorum* barks and leaves were submitted to off-line ESI-MS analysis of alkaloids. The lyophilised extracts were dissolved in methanol:water (1:1) and 100 mL of trifluoroacetic acid (1.0 mg/mL), filtered through a 0.2 mm size pore membrane and introduced into the mass spectrometer using a syringe pump. The spectra were obtained in the positive ionisation mode after using a triple quadruple Micromass^® ^Liquid Chromatograph Mass Spectrometer model Quattro Micro^TM^ API setting the capillary voltage at 2,300 V, the cone voltage at 60 V and the source at 100ºC. Each spectrum was produced by accumulation data over 1 min.

*Isolation of alkaloids from the stem bark extracts* - The neutral (0.69 g) and basic (0.36 g) fractions from the methanolic crude extract were subjected to a silica gel column chromatography, eluted with CHCl_3_ and followed by CHCI_3 _with increasing amounts of methanol. Further purification by preparative TLC was performed using a silica gel 60 GF_254_as the stationary phase and CHCl_3_:CH_2_Cl_2_:ethyl acetate:MeOH 4.5:4:1:0.5 (v/v) as eluents in an ammonium hydroxide (NH_4_OH) atmosphere (40 mg of catechin equivalents per plate). Two indole alkaloids, compounds 3 and 5, were isolated.

Compound 3, identified by ESI-MS at *m/z *327.3 [M + H] with daughter ions at *m/z* 251.0, *m/z* 175 and*m/z* 98.8, was previously described in the stem barks of*A. ramiflorum* and its structure was established by uni and bidimensional nuclear magnetic resonance (NMR). MS and comparison with the literature values confirmed compound 3 as 10-methoxygeissoschizol (Marques et al. 1996, Tanaka et al. 2007).

Compound 5 [α]^25^_D _0.0° (c = 1.0 mg/mL, EtOH) was identified by infrared (IR) (KBr,ν_max_ cm^-1^), 3345 (N-H and O-H), 1731 (COOCH_3_), ultraviolet (UV) λ (EtOH, Max) nm (log ε), 224 (4.7), 280 (4.14), 289 (4.08), electron impact (EI)/MS 70 eV, *m/z *(%), 354 [M+., 98], 323 (30), 251 (100), 249 (30), 169 (50), ESI-MS at *m/z*355.3 [M + H] and daughter ions at *m/z* 353.0 and*m/z* 279, ^1^H and ^13^C NMR (Table I). Compound 5 has not been previously described in *A. ramiflorum and* its structure was established by uni and bidimensional NMR and MS. By comparison with literature values compound 5 was (±-(*E*)-isositsirikine (Kan et al. 1981).

**TABLE I:** Antimalarial activity of *Aspidosperma ramiflorum*extracts and fractions in mice infected with *Plasmodium berghei* treated during three consecutive days by gavage

Oral treatment with	Dose (mg/kg)	Parasitaemia reduction at day 10^*a*^	Mice survival in days (increase)^*b*^
Plant extracts			
Neutral precipitate	250	66	26 ± 6 (4)
	500	53	29 ± 1 (7)^*c*^
Nonsoluble	250	16	29 ± 1 (7)^*c*^
	500	22	22 ± 1(0)
Chloroquine	20	100	> 30 (> 8)^*c*^
Control	0	0	22 ± 2

*a*: parasitaemia reduction in relation to untreated mice. Compounds inhibiting ≤ 30% were considered inactive, 30-50% as partially active and ≥ 50% as active; *b*: mice survival increase in days after drug treatment compared to survival of nontreated controls; *c*: significant differences in animal survival (p ≤ 0.05) by Mann-Whitney *U*test.

*Isolation of alkaloids from the leave extracts* - The basic alkaloid fraction (0.35 g) was placed on a silica gel column, eluted with CHCl_3_followed by CHCI_3 _with increasing amounts of MeOH and was further purified using a preparative TLC silica gel 60 GF_254_ as the stationary phase and then eluted with CHCl_3_:CH_2_Cl_2_:ethyl acetate:MeOH 4.5:4:1:0.5 (v/v) in an NH_4_OH atmosphere (40 mg of extract/per plate). The steps permitted the isolation of compound 2, an indole alkaloid.

Compound 2 [α]^25^_D _+ 58.5º (c = 1.0 mg/mL, EtOH) was identified by ESI-MS at *m/z *467.3 [M + H] with daughter ions at*m/z* 281.3 and *m/z* 174.0 as described before in stem barks (Marques et al. 1996). Its structure was established by uni and bidimensional NMR and MS data that confirmed its identity (2) as ramiflorine B by comparison with previous data (Tanaka et al. 2007).

*Tests with plant materials against P. falciparum blood parasites in vitro* - The activity of the *A. ramiflorum* extracts and fractions was evaluated against *P. falciparum* blood parasites [clone W2, chloroquine (CQ)-resistant], which were cultured as previously described (Trager & Jensen 1976) with modifications (de Andrade-Neto et al. 2004). The freshly sorbitol synchronised ring stages (Lambros & Vanderberg 1979) were immediately incubated with the test compounds at various concentrations that were previously solubilised in 0.05% dimethyl sulfoxide (DMSO) (v/v). Each test was performed in triplicate and the results were compared with the control cultures in complete medium with no drugs. CQ was used in each experiment as an antimalarial control. The compounds' effects were measured through the [^3^H]-hypoxanthine incorporation assay (Desjardins et al. 1979) and by the immunoenzymatic test, using specific monoclonal antibodies to a parasite protein histidine and alanine-rich protein (HRPII) that were commercially acquired (MPFM ICLLAB-55A^®^ and MPFG55P ICLLAB^®^, USA) (Noedl et al. 2002).

For the [^3^H]-hypoxanthine assay, the parasites were maintained at least four days in medium without hypoxanthine and adjusted to 1% parasitaemia and 1% haematocrit. The levels of [^3^H]-hypoxanthine incorporated into the parasites were measured using a beta counter (PerkinElmer, EUA). For the anti-HRPII test, parasitaemia was adjusted to 0.05% and the haematocrit to 1.5%; binding of the HRPII antibodies was quantified at 450 nm using a spectrophotometer (SpectraMax340PC^384^, Molecular Devices).

The half-maximal drug inhibitory concentration (IC_50_) was estimated by curve fitting using software from the OriginLab Corporation (USA) and comparing to the parasite growth in the drug-free medium.

*Cytotoxicity tests using immortalised or primary cells* - The cytotoxicity of the plant extracts and fractions was evaluated in a human hepatoma cell line (HepG2) and a monkey kidney cell line (BGM) using cells cultured in 75-cm^2^ sterile flasks containing RPMI-1640 medium (supplemented with 10% heat-inactivated foetal calf serum and 40 mg/L gentamicin) under a 5% CO_2_atmosphere at 37ºC. When confluent, the cell monolayer was washed with culture medium, trypsinised, distributed in a flat-bottomed 96-well plate (5 × 10^3^ cells/well) and incubated for 18 h at 37ºC for cell adherence (Denizot & Lang 1986). The compounds (20 µL), at various concentrations (1,000-1 µg/mL), were placed in the 96-well plates, incubated with the cultured cells for 24 h under a 5% CO_2_atmosphere at 37ºC and then the 3-(4,5-dimethylthiazol-2-yl)-2,5-diphenyltetrazolium bromide (MTT) solution (5 mg/mL; 20 µL/ well for 3 h) was used to evaluate the mitochondrial viability. The supernatants were carefully removed and 100 µL DMSO was added to each well and mixed to solubilise the formazan crystals. The optical density was determined at 570 nm and 630 nm (background) (SpectraMax340PC^384^). The cell viability was expressed as the percentage of the control absorbance in the untreated cells after subtracting the appropriate background.

Cytotoxicity was also tested using normal peripheral blood mononuclear cells (PBMC) isolated from healthy individuals by Ficoll-Histopaque (Sigma-Aldrich) gradient centrifugation (Panda & Ravindran 2013). The cells were washed twice at 100 *g* in RPMI-1640 (Sigma-Aldrich) and resuspended in 2 mL of RPMI supplemented with 10% heat-inactivated foetal bovine serum. The viable PBMCs cells were counted using the Trypan blue exclusion test, plated in 96-well cell culture plates at a final concentration of 3 × 10^6^cells/mL and incubated with the compounds (20 µL) at various concentrations (≤ 1,000 µg/mL) for 24 h at 37ºC. The MTT solution (5 mg/mL; 20 µL/ well) was added to evaluate the cell mitochondrial viability as described above. The minimum lethal dose for 50% (MLD_50_) of the cells was determined as previously described (Madureira et al. 2002). The selectivity index (SI) was calculated as the ratio between the activity and the cytotoxicity.

*Antimalarial tests against P. berghei in mice* - The drug suppressive test was performed as previously described (Peters 1965) with some modifications (Carvalho et al. 1991). Adult Swiss outbred mice (20 ± 2 g weigh) were inoculated by the intraperitoneal route with 1 × 10^5^ red blood cells infected with*P. berghei*, the NK65 strain. Groups of 20-30 infected mice were kept together for 2-24 h, then were divided randomly in groups of up to six animals per cage and were treated by gavage for three consecutive days with 25 mg/Kg or 50 mg/Kg of the test compounds diluted in DMSO 3% (v/v). Two control groups were used and either received CQ (20 mg/kg, diluted in water) or the drug vehicle. Smears prepared from the tail blood of the mice on days 5-10 post-infection were methanol-fixed, stained with Giemsa and examined microscopically. Parasitaemia was evaluated and the percent inhibition of the parasite growth was calculated in relation to the untreated control group, which was considered 100% growth.

## RESULTS

The results of the in vitro activity of the extracts from the stem barks and the leaves of *A. ramiflorum *against the blood cultures of *P. falciparum *(W2 clone, CQ-resistant) are shown in Table II. The compounds with an IC_50_ below 10 µg/mL were considered active. All but the aqueous extracts were active, particularly the acid, the NP and the basic bark crude extracts, which showed IC_50_values between 0.5-3.8 µg/mL. Both the hypoxanthine and the anti-HRPII assays provided similar results. The methanolic and acetone extracts from the leaves showed IC_50_ values of 1.4 and 4.0 µg/mL, respectively. The pure substances (5, 3 and 2) isolated from the bark fractions (neutral and basic) were active. Compound 5 had the lowest IC_50_ (0.3 µg/mL). All of these extracts were rich in indole alkaloids.

**TABLE II:** In vitro activity of *Aspidosperma ramiflorum*compounds tested against blood forms of *Plasmodium falciparum *[W2 clone, chloroquine (CQ)-resistant parasites], cytotoxicity [minimum lethal dose for 50% (MLD50)] to a human hepatoma cell line (HepG2), a monkey kidney cell line (BGM) and human peripheral blood mononuclear cells (PBMC) and selectivity indexes (SI)

Compounds^*a*^	MLD_50_ (µg/mL)			IC_50_ (µg/mL)		SI		
	BGM	HepG2	PBMC	Hypoxanthine	Anti-HRPII	BGM	HepG2	PBMC
Bark extracts								
Acid	388 ± 100	155 ± 2	512 ± 34	3.8 ± 1.2	2.5 ± 1.2	138	41	135
Neutral	31 ± 2	35 ± 5	NT	0.9 ± 0.1	0.7 ± 0.5	34	39	NT
Basic	32 ± 0	31 ± 0	11 ± 4	0.5 ± 0.2	0.8 ± 0.5	64	62	22
Neutral precipitate	30 ± 1	45 ± 0	13 ± 4	0.7 ± 0.4	0.7 ± 0.4	43	64	19
Methanolic residue	88 ± 16	80 ± 43	25 ± 6	1.7 ± 1.6	1.5 ± 0.9	52	47	15
Nonsoluble	621 ± 120	173 ± 7	149 ± 36	3.1 ± 0.6	3.1 ± 1.8	200	56	48
Aqueous	> 1,000	> 1,000	> 1,000	> 50	> 50	Inative	Inative	Inative
Pure substances								
5 (isositsirikine)	28 ± 7	34 ± 1	NT	0.3 ± 0.1	0.2 ± 0.0	93	113	NT
3 (10-MG)	8 ± 3	5 ± 1	NT	1.0 ± 0.9	0.4 ± 0.3	Toxic	Toxic	NT
2 (ramiflorine B)	5 ± 2	5 ± 1.7	NT	1.2 ± 0.4	0.9 ± 0.9	Toxic	Toxic	NT
Leave extract								
Methanolic	98 ± 22	137 ± 18	64 ± 3	3.6 ± 1.3	1.4 ± 0.7	27	38	18
Acetone	37 ± 6	67 ± 12	32 ± 8	1.7 ± 1.7	1.4 ± 0.4	22	39	19
CQ	457 ± 22	398 ± 12	222 ± 73	0.100 ± 0.21	0.0 7± 0.10	4,570	3,980	2,220

**Fig. 3:**
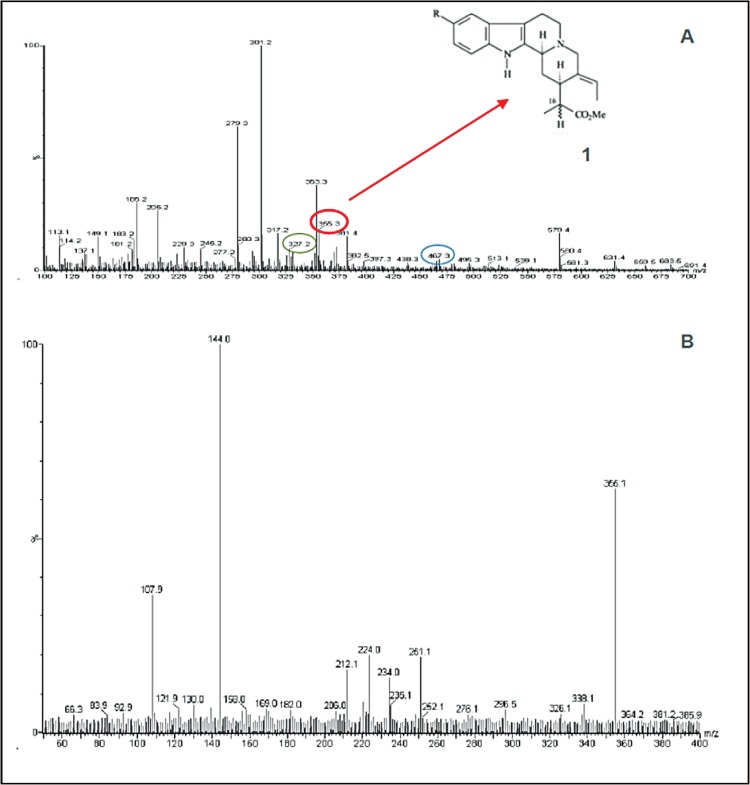
off-line ESI-MS of crude extract from stem bark of*Aspidosperma ramiflorum*. Ion at *m/z*467 corresponding to ramiflorine A (1) and ramiflorine B (2), ion at *m/z *327 corresponding to 10-methoxy-geissoschizol (3) and ion at *m/z *355 corresponding to β-yohimbine (4) and isositsikine (5).

*a*: the steps for extract biofractionation are summarised in Fig. 3; HRPII: histidine and alanine-rich protein; IC_50_: dose inhibiting 50% parasite growth evaluated in three-four experiments for each test; MG: methoxy-geissoschizol; NT: not tested.

The plant showed no toxicity towards the HepG2 and BGM cell lines and showed high MLD_50_ values for most of the extracts and fractions. The acid and nonsoluble extracts were toxic to the normal cells (BGM) (Table II). When evaluated against freshly isolated human PBMC, the MLD_50_ values were lower for most of the extracts and fractions tested, except for the acid, the aqueous and the acetone extracts, which had similar MLD_50_ values in relation to the immortalised cell line. The SI of compound 5 was the highest (SI = 100), but substances 3 and 2 had SI values below 10, which were indicative of toxicity; the other purified compounds had SI values above 22 (Table II).

Two plant fractions were tested in vivo: the neutral and the precipitate fractions from the stem barks. The NP fraction reduced the *P. berghei*parasitaemia to 53% on day 10 in relation to the nontreated control mice. However, both of the extracts increased the survival of the mice (Table I). The other extracts and purified compounds were not tested in vivo due to the insufficient amounts available.

The mass peaks observed in the chemical characterisation of the crude extract from the barks of *A. ramiflorum* used in the ESI-MS analysis are shown inFig. 3. A new molecule was identified, isositsirikine (5) at *m/z *355 [M + H]+ (Table III). The other isolated compounds were ramiflorine A (1) and B (2) at *m/z *467 [M + H]+, 10-methoxy-geissoschizol (3) at 327 [M + H]+ and β-yohimbine (4) and these compounds correspond to compounds previously described in the species (Marques et al. 1996, Tanaka et al. 2007). All of the fractions from the stem bark had similar mass spectra profiles.

**TABLE III:** 1H (300 MHz) and 13C (75 MHz) nuclear magnetic resonance (NMR) data of compound (5) in CDCl3 as solvent and tetramethylsilane used as internal reference, chemical shifts (d, ppm) and coupling constants (*J*, Hz)

(5)/CDCl_3_		
Compound	*δ* ^13^C	^1^H *J *(Hz)
2	133.2	-
3	49.7	4.30 *ls*
5α	51.3	3.12 *m*
β	-	3.30 *m*,* J *= 13.0; 7.3
6α	17.6	2.65 *dd*,* J *= 15.0; 4.8
β	-	3.00 *m*
7	107.7	-
8	127.6	-
9	117.9 1	7.52 *t*, *J *= 7.50
10	21.6	7.12 *d*, *J *= 7.50
11	119.5	7.12 *t*, *J *= 7.50
12	111.3	7.48 *d*, *J *= 7.50
13	ND	-
14α	30.0	2.26 *m*
β	-	2.20 *m*, *J *= 5.00
15	32.5	3.10 *m*
16	52.3	2.52 *m*
17R	61.9	3.55 *m*
17S	-	3.50 *m*
18	13.3	1,67 *d*,* J *= 6.0; 1.1
19	121.6	5.64 *q*,* J *= 6.0
20	137.7	-
21α	52.1	3.54 *m*
β	-	2.93 *m*
-OCH_3_	52.7	3.80 *s*
C = 0	175.4	-
N_(1)-_H	ND	8.75 *s*
OH	ND	2.00 s

ND: not determined.

## DISCUSSION

The chemical analysis of the *A. ramiflorum *crude extract showed the mass spectra profiles of two compounds that were similar to what had been described before in the plant leaves (Marques et al. 1996). Their mass peaks corresponded to the lead alkaloid compounds ramiflorine A (1) and ramiflorine B (2) at *m/z *467 [M + H]+. Using preparative TLC, the bioactive fractions from the stem bark (neutral and basic) and the leaves (basic) were chromatographed on a silica gel column to generate purified fractions and two previously described compounds in *A. ramiflorum*were identified, the indole alkaloids ramiflorine B (2) and 10-methoxy-geissoschizol (3), whereas the compound isositsirikine (5) was reported here for the first time in the species.

The structures of 2, 3 and 5 were established in this study using uni and bidimensional NMR and the values of the MS analysis in comparison with previous literature data (Kan et al. 1981, Marques et al. 1996, Tanaka et al. 2007).

The mass spectrum of 5 gave a molecular ion at *m/z* 354 (M+, EI/MS 70 eV) as well as peaks at *m/z* 323, 251, 249 and 169, which are typical of the corynanthine skeleton. The IR and UV spectra showed absorption bands characteristic of a 10-methoxyindole chromophore (Marques et al. 1996). The ^1^H and ^13^C NMR spectra showed downfield signals at δH 7.52 (1H, t, J= 7.5 Hz), 7.12 (1H, d, J = 7.5 Hz), 7.12 (1H, t, J = 7.5 Hz) and 7.48 (1H, d, J = 7.4 Hz), which correlate with the respective carbons at δ_C_ 117.9 (C-9), 121.6 (C-10), 119.5 (C-11) and 111.3 (C-12) (heteronuclear single quantum coherence). Additional signals were detected from four quaternary carbons, one with downfield signals at δ_C_175.4 (COOCH_3_) and the other four at δ_C_ 133.2 (C-2), 107.7 (C-7), 127.6 (C-8) and 137.7 C-20). ^1^H NMR spectrum also showed the presence of an ethylidene side chain methyl group (C-19) at δ_H_ 1.67 (3H, d, *J* = 6.0 Hz) and an olefinic proton (C-18) at δ_H_ 5.64 (1H, q, *J* = 6.0 Hz) and at δ_H_ 4.30 (1H, sl) that correlated with δ_C _49.7. The H-3 α-configuration was confirmed by comparison with the chemical shift of the protons of similar compounds described in the literature (Kan et al. 1981). The^1^H and ^13^C NMR data of alkaloid 5 were similar to those reported for a compound known as isositsirikine (Kan et al. 1981). We suggest that the presence of the indole alkaloid skeleton (3 and 5) explains the decrease in the cytotoxicity of the compounds and the consequent increase in the SI values; indeed, the dimeric ramiflorine B (2) was more cytotoxic than 3 and 5.

The results of the values of the MLD_50_ in the PBMC nonimmortalised cells showed a slightly higher cytotoxicity in comparison with the immortalised cells for some samples, which can be attributed to the high multiplication rates of the other cell lines.

It was only possible to test the in vivo antimalarial activity of the neutral and precipitate extracts from the stem barks in mice and both extracts increased survival of the mice, but whether this was due to the presence of the alkaloids in*A. ramiflorum* having anti-inflammatory and antioxidant activities (Barbosa-Filho et al. 2006) needs to be clarified. It is also necessary to study the in vivo activity of isositsirikine (compound 5), the most active compound against *P. falciparum*in vitro, which was demonstrated for the first time in the present study.

The pharmacokinetic profile of the active extracts and the alternative routes of drug administration were not determined and should be further evaluated. Other biological processes, such as drug metabolism and/or drug excretion may act on the compounds' activities in the body and thus, decline their amounts in an exponential manner (Alavijeh et al. 2005) thereby altering the in vivo activity of the plant products.

## References

[B1] Adebayo JO, Krettli AU (2011). Potential antimalarials from Nigerian plants: a
review. J Ethnopharmacol.

[B2] Aguiar ACC, da Rocha EMM, de Souza NB, França TCC, Krettli AU (2012). New approaches in antimalarial drug discovery and
development - A Review. Mem Inst Oswaldo Cruz.

[B3] Alavijeh MS, Chishty M, Qaiser MZ, Palmer AM (2005). Drug metabolism and pharmacokinetics, the blood-brain
barrier and central nervous system drug discovery. Neuro Rx.

[B4] Barbosa JM, Piuvezam MR, Moura MD, Silva MS, Lima KVB, da Cunha EVL, Fechine I, Takemura O (2006). Anti-inflammatory activity of alkaloids: a
twenty-century review. Rev Bras Farmacogn.

[B5] Bourdy G, Willcox ML, Ginsburg H, Rasoanaivo P, Graz B, Deharo E (2007). Ethnopharmacology and malaria: new hypothetical leads or
old efficient antimalarials?. Int J Parasitol.

[B6] Carvalho L, Brandão M, Santos D, Lopes J, Krettli A (1991). Antimalarial activity of crude extracts from Brazilian
plants studied in vivo in Plasmodium berghei-infected mice and in vitro against
Plasmodium falciparum in culture. Braz J Med Biol Res.

[B7] Coutinho JP, Aguiar ACC, dos Santos PA, Lima JC, Rocha MGL, Santana AEG, Pereira MM, Krettli AU (2013). Aspidosperma (Apocynaceae) plant cytotoxicity and
activity towards malaria parasites. Part I: Aspidosperma nitidum (Benth) used as a
remedy to treat fever and malaria in the Amazon. Mem Inst Oswaldo Cruz.

[B8] Cui L, Su XZ (2009). Discovery, mechanisms of action and combination therapy
of artemisinin. Expert Rev Anti Infect Ther.

[B9] Cunha AC, Chierrito TP, Machado GM, Leon LL, da Silva CC, Tanaka JC, de Souza LM, Gonçalves RA, de Oliveira AJ (2012). Anti-leishmanial activity of alkaloidal extracts
obtained from different organs of Aspidosperma ramiflorum. Phytomedicine.

[B10] de Andrade VF, Brandão MGL, Oliveira FQ, Casali VWD, Njaine B, Zalis MG, Oliveira LA, Krettli AU (2004). Antimalarial activity of Bidens pilosa L. (Asteraceae)
ethanol extracts from wild plants collected in various localities or plants
cultivated in humus soil. Phytother Res.

[B11] de Andrade VF, Pohlit AM, Pinto ACS, Silva ECC, Nogueira KL, Melo MRS, Henrique MC, Amorim RCN, Silva LFR, Costa MRF, Nunomura RCS, Nunomura SM, Alecrim WD, Alecrim MGC, Chaves FCM, Vieira PPR (2007). In vitro inhibition of Plasmodium falciparum by
substances isolated from Amazonian antimalarial plants. Mem Inst Oswaldo Cruz.

[B12] de Ridder S, van der Kooy F, Verpoorte R (2008). Artemisia annua as a self-reliant treatment for malaria
in developing countries. J Ethnopharmacol.

[B13] Denizot F, Lang R (1986). Rapid colorimetric assay for cell growth and survival.
Modifications to the tetrazolium dye procedure giving improved sensitivity and
reliability. J Immunol Methods.

[B14] Desjardins RE, Canfield CJ, Haynes JD, Chulay JD (1979). Quantitative assessment of anti-malarial activity in
vitro by a semiautomatated microdilution technique. Antimicrob Agents Chemother.

[B15] Frausin G, Hidalgo AF, Lima RB, Kinupp VF, Ming LC, Pohlit AM, Milliken W (2015). An ethnobotanical study of anti-malarial plants among
indigenous people on the upper Negro River in the Brazilian Amazon. J Ethnopharmacol.

[B16] Kan C, Kan SK, Lounasmaa M, Husson HP (1981). Trapping of intermediates in the interconversion of
heteroyohimbine alkaloids. Acta Chem Scand.

[B17] Krettli AU, Adebayo JO, Krettli LG (2009). Testing of natural products and synthetic molecules
aiming at new antimalarials. Curr Drug Targets.

[B18] Krettli AU, Andrade VF, Brandão MGL, Ferrari WMS (2001). The search for new antimalarial drugs from plants used
to treat fever and malaria or plants randomly selected: a Review. Mem Inst Oswaldo Cruz.

[B19] Lambros C, Vanderberg JP (1979). Synchronization of Plasmodium-falciparum erythrocytic
stages in culture. J Parasitol.

[B20] Madureira MC, Martins AP, Gomes M, Paiva J, da Cunha AP, Rosário V (2002). Antimalarial activity of medicinal plants used in
traditional medicine in S. Tomé and Príncipe islands. J Ethnopharmacol.

[B21] Marques MFS, Kato L, Leitão HF, Reis FAM (1996). Indole alkaloids from Aspidosperma
ramiflorum. Phytochemistry.

[B22] Noedl H, Wongsrichanalai C, Miller R, Myint K, Looareesuwan S, Sukthana Y, Wongchotigul V, Kollaritsch H, Wiedermann G, Wernsdorfer W (2002). Plasmodium falciparum: effect of anti-malarial drugs on
the production and secretion characteristics of histidine-rich protein
II. Exp Parasitol.

[B23] Oliveira AB, Dolabela MF, Braga FC, Jácome RL, Varotti FP, Póvoa MM (2009). Plant-derived antimalarial agents: new leads and
efficient phythomedicines. Part I. Alkaloids. An Acad Bras Cienc.

[B24] Oliveira DR, Krettli AU, Aguiar AC, Leitão GG, Vieira MN, Martins KS, Leitão SG (2015). Ethnopharmacological evaluation of medicinal plants used
against malaria by Quilombola communities from Oriximiná, Brazil. J Ethnopharmacol.

[B25] Panda SK, Ravindran B (2013). In vitro culture of human PBMCs. Bio Protoc.

[B26] Peters W (1965). Drug resistance in Plasmodium berghei Vincke and Lips,
1948. I. Chloroquine resistance. Exp Parasitol.

[B27] Ridley RG (2002). Medical need, scientific opportunity and the drive for
antimalarial drugs. Nature.

[B28] Rocha e Silva LF, Montoia A, Amorim RC, Melo MR, Henrique MC, Nunomura SM, Costa MR, Andrade VF, Costa DS, Dantas G, Lavrado J, Moreira R, Paulo A, Pinto AC, Tadei WP, Zacardi RS, Eberlin MN, Pohlit AMLF (2012). Comparative in vitro and in vivo antimalarial activity
of the indole alkaloids ellipticine, olivacine, cryptolepine and a synthetic
cryptolepine analog. Phytomedicine.

[B29] Tanaka JCA, Silva CC, Ferreira ICP, Machado GMC, Leon LL, Oliveira AJB (2007). Antileishmanial activity of indole alkaloids from
Aspidosperma ramiflorum. Phytomedicine.

[B30] Torres ZES, Silveira ER, Silva LFR, Lima ES, de Vasconcellos MC, Uchoa DEA, Braz R, Pohlit AM (2013). Chemical composition of Aspidosperma ulei Markgr. and
antiplasmodial activity of selected indole alkaloids. Molecules.

[B31] Trager W, Jensen JB (1976). Human malaria parasites in continuous
culture. Science.

[B32] White NJ (2008). Qinghaosu (artemisinin): the price of
success. Science.

[B33] WHO - World Health Organization (2014). Malaria.

[B34] Willcox ML, Bodeker G (2004). Traditional herbal medicines for malaria. BMJ.

